# Methylation-Based Therapies for Colorectal Cancer

**DOI:** 10.3390/cells9061540

**Published:** 2020-06-24

**Authors:** Klara Cervena, Anna Siskova, Tomas Buchler, Pavel Vodicka, Veronika Vymetalkova

**Affiliations:** 1 Department of Molecular Biology of Cancer, Institute of Experimental Medicine, Videnska 1083, 14 200 Prague, Czech Republic; klara.cervena@iem.cas.cz (K.C.); anna.siskova@iem.cas.cz (A.S.); pavel.vodicka@iem.cas.cz (P.V.); 2 Institute of Biology and Medical Genetics, First Faculty of Medicine, Charles University, Albertov 4, 128 00 Prague, Czech Republic; 3 Department of Oncology, First Faculty of Medicine, Charles University and Thomayer Hospital, Videnska 800, 140 59 Prague, Czech Republic; tomas.buchler@ftn.cz; 4 Biomedical Centre, Faculty of Medicine in Pilsen, Charles University, Alej Svobody 76, 323 00 Pilsen, Czech Republic

**Keywords:** colorectal cancer, methylation, DNMT inhibitors, therapy

## Abstract

Colorectal carcinogenesis (CRC) is caused by the gradual long-term accumulation of both genetic and epigenetic changes. Recently, epigenetic alterations have been included in the classification of the CRC molecular subtype, and this points out their prognostic impact. As epigenetic modifications are reversible, they may represent relevant therapeutic targets. DNA methylation, catalyzed by DNA methyltransferases (DNMTs), regulates gene expression. For many years, the deregulation of DNA methylation has been considered to play a substantial part in CRC etiology and evolution. Despite considerable advances in CRC treatment, patient therapy response persists as limited, and their profit from systemic therapies are often hampered by the introduction of chemoresistance. In addition, inter-individual changes in therapy response in CRC patients can arise from their specific (epi)genetic compositions. In this review article, we summarize the options of CRC treatment based on DNA methylation status for their predictive value. This review also includes the therapy outcomes based on the patient’s methylation status in CRC patients. In addition, the current challenge of research is to develop therapeutic inhibitors of DNMT. Based on the essential role of DNA methylation in CRC development, the application of DNMT inhibitors was recently proposed for the treatment of CRC patients, especially in patients with DNA hypermethylation.

## 1. Introduction

Human malignancies develop as a result of the accumulation of genetic and epigenetic changes. It is well known that both alterations can be observed not only in cancer cells but also in nonmalignant cells, even before tumor occurs. In addition, epigenetic changes have been identified as crucial mechanisms that underlie colorectal cancer (CRC) development and progression [1,2,3,4].

DNA methylation is the most broadly investigated epigenetic alteration (reviewed in [5]). Except for adenine, cytosine, guanine, and thymine, the human genome comprises an additional base, 5-methyl-cytosine (5-mC). 5-mC can be transformed into thymine spontaneously or by deamination controlled enzymatically. Although the brain contains the highest levels of methylated DNA, it is estimated that 5-mC counts for less than 1% of nucleic acids [6]. This methylated form of cytosine is present almost exclusively in connection with CpG dinucleotides. Around 50% of genes in mammal genomes have short (~1 kb long) CpG-rich regions known as CpG islands (CGIs) spread in DNA and are predominantly associated with transcription start sites and promoters [7]. The majority of the cytosines in the CpG sites are methylated in human somatic cells. However, this number alters between diverse tissues and pathological status and is lower with increased age [8].

## 2. The Source of Methylation Alterations

DNA methylation seems to be caused by exposure to different natural stimulants and dietary factors [9,10]. First, aging has been shown to correlate with aberrant DNA hypermethylation [11]. Additionally, chronic inflammation has been associated with the induction of aberrant DNA hypermethylation [12,13,14,15]. It has been determined that patients with chronic inflammation in their bowels are at increased risk of CRC. Cigarette smoking was noted to induce DNA hypermethylation in vitro, and it has been verified in vivo, where high levels of DNA methylation in nonmalignant tissues from the esophagus of smokers were observed [16]. Another inducer of differences in DNA methylation has been ascribed to estrogen treatment in cultured mammary epithelial cells [17]. Dietary factors may affect one-carbon metabolisms, including B vitamins, which are coenzymes in one-carbon metabolisms (vitamins B_2_, B_6_, and B_12_) and might be considered as the regulators of DNA methylation [18]. DNA hypomethylation has been observed during low folate supply and without vitamin B_12_ in the diet [19,20].

The connection between DNA methylation and aging has been pointed out in the last few decades of research. DNA methylation is an endogenous source of mutations and thus contributes to mutation burden, causing DNA damage and leading to apoptosis and the aging or death of the organism [21]. Several authors have hypothesized that an epigenetic drift or clock associated with aging exists, and they have suggested that DNA methylation is associated with longevity [22,23,24]. Moreover, Fraga et al. [25] identified, by examining the differences in DNA methylation profiles of monozygotic twins, that younger twins have identical methylomes, while older twins have remarkably different methylomes.

During the development and progression of CRC, several age-related DNA methylation changes can be observed that affect the mRNA expression of genes associated with the transition from adenoma to CRC. While global hypomethylation is characteristic during aging, among age-dependent hypermethylated genes, many tumor suppressor genes have been reported (reviewed in [22,26].

DNA methylation also plays an important role in the regulation of inflammatory genes in many diseases, including CRC. Many studies have presented that methylation of distinct genes (such as *SOCS3* [27], *PRKCDBP* [28], *CYP1B1* [29], *PTX3* [30], *FXR* [31], and *VDR* [32]) is associated with inflammatory conditions, dysplasia, and malignant transformations, suggesting that these modifications are involved in inflammatory-induced carcinogenesis [12,33,34].

In colitis-associated CRC samples, the expression of *DNMT1* was significantly higher than in sporadic CRC tumors, suggesting an increased level of DNA methylation in inflammatory tissues [35]. In addition, hypermethylation of the *ITGA4, TFPI2,* and *VIM* gene promoters was noticed in inflammatory tissues of the colon, which may pose a higher risk to the development of colitis-associated CRC [36].

## 3. Functions of DNA Methylation

This epigenetic alteration is crucial for retroviral elements silencing, regulation of tissue-specific gene expression, genomic imprinting, and the inactivation of the X chromosome. Even though aberrant DNA methylation correlates with transcription silencing, the basic mechanisms are not necessarily the same as gene promoters, gene bodies, or repeated sequences.

Most of the CGIs remain unmethylated in somatic cells; however, some silenced genes contain methylated promoter CGIs. Those are generally limited to genes with long-lasting stabilization of suppressed status such as imprinted genes, genes located on the inactive X chromosome, and genes expressed only in germ cells.

As stated before, many CGIs are situated in promoters, but CGIs can be located within the gene body and in desserts [37]. The majority of gene bodies lack CpGs; however, they are broadly methylated and have multiple repetitive and transposable elements. Gene body methylation is generally a feature of transcribed genes [38]. Methylation of the CpG sites within exons is the main purpose of C→T transition mutation origin and is responsible for about 30% of all disease-causing mutations in the germline [39,40]. Exons have been shown to be more methylated than introns, and transitions in the level of methylation appear at the boundaries of exons and introns, which may indicate the importance of methylation in the regulation of splicing [41,42]. The nucleosome position data throughout the genome suggest that exons also evince a higher level of nucleosome occupancy in contrast to introns [43], and DNA methylation is higher in DNA comprising nucleosome than in flanking DNA [44].

Methylation in repeating regions, such as centromeres, is important for chromosomal stability [45] (e.g., chromosomal segregation during mitosis) as it might repress the expression of transposable elements [46].

Methylated CGIs at transcriptional start sites (TSSs) are not able to establish transcription after assembling the DNA into nucleosomes [47,48,49]. It has been shown that methylation at CGI within the promoter represses gene expression. However, most of the genes have at least two TSSs, which likely to represent alternative promoters, and their methylation hampers the interpretation of experiments studying the expression linked to methylation [50,51]. Nevertheless, the question of whether repressed status or methylation comes first has long been a topic of discussion in this area. Genes with CGI in their promoters, which are already repressed by Polycomb complexes, are more likely to be methylated than other genes in cancer: thus, the repressed state precedes methylation [52,53,54,55]. Polycomb proteins repress gene expression by histone modification, especially during development and differentiation [56] and silence tumor suppressor genes [57]. The mechanism of alternative gene silencing by Polycomb complex is through the trimethylation of histone H3, chromatin compaction, and regulation of H2A by monoubiquitylation [58,59].

Therefore, it seems that a suppressed state preceding DNA methylation is understood as a fundamental mechanism. However, the results are still not clear. The evidence regarding the timing of DNA methylation suggests that methylation adds another level of stability to epigenetic states. However, tissue-specific methylation changes exist in “shores” and “shelves” surrounding them [60]. These regions bear information that is very important for mediating the control gene expression [60,61]. For example, CGI shores and shelves show higher variation in different cancers, and this site-specific methylation should be kept in mind during the analyses. Differential methylation is not limited to the CGIs but also applies to CpG regions, like at enhancers [62].

There is also a significant fraction of genes that do not display consistency with the methylation-induced repression of transcription. Recently, Spainhour et al. [63] identified that these discrepancies may have several reasons. The authors observed that methylation at particular CpG sites was correlated with corresponding gene expression in the same direction in all cancer types, either with positive or negative correlation. The authors also hypothesized that the connection between DNA methylation and mRNA expression is tissue-independent. In addition, CpG sites close to each other often experience the same trend of correlation with mRNA expression. Another fact influencing the correlation between DNA methylation and gene expression is the position of the CpG site. In the same study of Spainhour [63], the methylation at the CpG site, right before the TSS, was negatively correlated with gene expression, while the methylation at the CpG site after the transcription end site (TES) was positively correlated with expression. This observation points to the fact that methylation of CpG sites at different locations in the gene body displays distinct functions and regulatory effects.

## 4. DNA Methylation and DNA Methyltransferases (DNMTs)

DNA methylation is an essential process of regulating gene expression without altering the genetic information and is mediated by DNA methyltransferases (DNMTs) that use *S*-adenosyl-l-methionine as a source of the methyl group. DNMTs can be categorized according to their function into three groups: most abundant DNMT1, de novo DNMTs (DNMT3A and DNMT3B), and DNMT2 (with methylation activity for tRNA) [64,65]. DNMT1 is responsible for maintaining DNA methylation and catalyzes the attachment of a methyl group to a CpG dinucleotide on DNA strands during replication [66]. DNMT3A and DNMT3B are essential for de novo methylation of the genome, to keep a cell’s specific methylation profile, and also for methylation of newly integrated retroviral sequences [67,68].

The process of transcriptional repression also requires the so-called “reader” molecules, known as methyl-binding proteins (MBPs) and their corepressors, such as histone deacetylases (HDAC1 and HDAC2), Sin3A, and Mi-2 [69]. The role of MBPs is to bind symmetrically to the methylated CpG to attract different members of the chromatin remodeling complex [70]. MBPs contain several proteins that are involved in chromatin remodeling and can be classified into three families based on the functional domains: MBPs with methyl binding domains (MBDs), MBPs that recognizes the methylated DNA according to the Zinc finger motifs, and MBPs with ability to bind methylated DNA using the Set- and RING-associated (SRA) domain [71].

The opposite action to DNA methylation is the elimination of methyl groups, known as the demethylation process. This process involves multiple mechanisms, such as oxidative demethylation of 5-mC, enzymatic removal of the methyl group, or even the base excision repair of the DNA molecule. The demethylation process is facilitated by ten–eleven translocation proteins (TET), the family of proteins that catalyzes the conversion of 5-mC to 5-hydroxycytosine, and subsequently, to cytosine [72]. To promote DNA demethylation, TET1 attaches to the CpG-rich regions in gene promoters and at transcription start sites (TSS) [73].

DNA methylation regulation is mainly facilitated through DNMT1 [74]. DNMT1 modifications are site-specific and enzyme-dependent [74,75]. DNMT1 is also modified by SUMOylation, a process where small ubiquitin modifying proteins (SUMO) are attached by covalent bonds to DNMT1 [76]. In addition, DNMTs can undergo acetylation, phosphorylation, methylation, or ubiquitination [77,78,79]. Moreover, polycomb (PcG) proteins can directly bind to DNMTs and thus regulate their activity [80].

DNMTs were also studied for their role in CRC pathogenesis. The overexpression of DNMT3B1 increased the number of colon tumors in APC Min/+ mice and enlarged the average size of colorectal adenomas, while the overexpression of DNMT3A1 displayed no effect. DNMT3B1 but not DNMT3A1 methylated the same genes in tumors and in nonmalignant tissues, suggesting that de novo DNMTs can also trigger the methylation and silencing of specific genes in a nonmalignant cell [81]. Interestingly, the overexpression of DNMT3B was associated with a CIMP-high in CRC [82,83]. DNMTs are rarely mutated in cancers. Kanai et al. [84] noticed that mutational changes in the *DNMT1* gene that lead to its inactivation in colon cancer cells resulted in changes in the specific DNA methylation profiles.

## 5. Alterations of DNA Methylation in the Cancer Genome

Aberrant DNA methylation is a hallmark of almost all cancer types. However, many methylation alterations are cancer-specific and appear early in the cancer genome [85], long before the malignant transition, and enlarge with progression [86]. DNA methylation of the promoter CGIs of tumor suppressor genes was suggested as an alternative mechanism in Knudson’s two-hit theory of tumor suppressor gene inactivation, and alongside the specific methylation profile, has been associated with the outcome of different cancer types [87].

The incidence of germline epigenetic features in connection with cancer development in humans is unique, and its presence has so far been found only in familial cancer syndrome. Chan et al. [88] analyzed germline hypermethylation of the *MSH2* gene in a three-generation family with hereditary nonpolyposis colorectal cancer (HNPCC) without evidence of DNA mismatch repair gene mutations. Despite the potential implications for genetic diagnosis, epigenetic inheritance in humans is an underestimated field, with a rare occurrence of uncovered cases.

Cancer genomes synchronously display global hypomethylation as well as gene-promoter-specific hypermethylation. Therefore, the discovery of hypo- and hypermethylated genes might identify new aspects crucial for cancer initiation and progression. In addition, several changes in DNA methylation have an impact on genes involved in carcinogenesis or drug response and represent a promising therapeutic target [89]. Despite that hypomethylation and hypermethylation show a close relationship to each other, they contribute separately to the CRC [90,91,92].

The carcinogenic process also involves the interactions between cancer cells and components of the surrounding microenvironment, consisting of the extracellular matrix, fibroblasts, endothelium, and vasculature-associated pericytes, as well as immune cells and occasional adipose cells [93]. Fibroblasts around cancer cells have been considered cancer-associated fibroblasts (CAFs). They differ phenotypically and functionally from normal fibroblasts (NFs) and provide cancer cells with nutrition and energy and stimulate the proliferation, invasion, and metastasis of cells [94,95]. CAFs achieve their activated phenotype in the tumor microenvironment through interactions with tumor cells or tumor-cell-derived biomolecules. CAFs represent the most numerous populations in the tumor stroma, contributing to carcinogenesis by excreting diverse factors (α-smooth muscle actin (α-SMA), fibroblast activation protein α (FAPα), and fibroblast specific protein-1 (FSP-1)) that regulate intercellular signaling in tumor cells, and mechanically restore cancer tissue [96,97,98,99]. The epigenetic programming in CAFs serves as a permanent change that stimulates tumor growth [100]. The restoration of the epigenetically silenced gene expressions in the CAF by 5-azacitidine resulted in reduced tumor progression [101]. In CRC, hypomethylation of chondroitin sulfate proteoglycan only occurs in the CAFs of tumor stroma, rather than in cancer cells [102]. DNA promoter hypomethylation may also exist in CAFs. Matsunoki et al. [103] observed that activation of *LINE-1* in CRC is due to its hypomethylated promoter.

## 6. Methylation-based Etiology in CRC

The role of the CpG island methylator phenotype (CIMP) in CRC was already postulated in 1999 by Toyota et al. [104]. The authors demonstrated that the *MLH1* gene was frequently inactivated by promoter methylation in the CIMP-positive group of CRC patients.

The majority of the sporadic microsatellite unstable CRC tumors show a CIMP-positive status, while CIMP is infrequent in microsatellite instable (MSI) HNPCC tumors [105,106]. In addition, around 30–40% of sporadic colon cancers located in the proximal colon exhibit CIMP-positive status compared to 3–12% of tumors located in the distal colon and rectal cancers [107,108,109,110,111,112]. Thus, CIMP is more common in tumors originating in the proximal colon, independent of the MSI status. The presence of CIMP has been correlated with *BRAF* mutations (*BRAF* V600E) in both microsatellite stable and MSI colon cancers [105,109,111,113]. CRCs with chromosomal instability (CIN) and CIMP have been shown to correlate inversely [114,115] and develop in two separate pathways [116].

DNA hypermethylation of several CIMP-associated genes has been observed during the early CRC stages [82]. In addition, promoter DNA hypermethylation was determined in the nonmalignant colon mucosa in the patients that had a predisposition to multiple serrated polyps, which are considered as the precursors of CIMP tumors [117]. Weisenberger et al. [105] reported that DNA hypermethylation of the *MLH1* gene associated with CIMP is the main mechanism for the evolution of sporadic MSI CRC. Several authors have indicated that *KRAS*-mutated colorectal tumors are in a close link with specific DNA methylation profiles [118,119,120,121,122]. Several years ago, CIMP-low (CIMP-L) status was significantly associated with *KRAS* mutations and the male gender, and it is independent of MSI status [118].

Based on these results, there were debates concerning the CRC subtypes classified due to their DNA methylation status. Many researchers believe that patient stratification based on molecular subtypes can help stratify individual patients for specific treatment.

## 7. CRC Molecular Subtypes in Therapy Outcome Prediction

CRC is a very heterogeneous disease. Each patient has a unique genetic and epigenetic background, and due to this heterogeneity, mortality and response to treatment vary. Thus, CRC can be categorized based on different molecular characteristics. The assessment of molecular subtypes is necessary for a better understanding of the CRC etiology [123], but also important in selecting proper therapy, predicting patient outcomes, and discovering risk factors associated with a particular subtype [124,125,126]. Historically, CRC classification was done primarily on clinical and pathological characteristics (like tumor localization, tumor stage, or degree of differentiation). In the last century, it has become clear that CRC can arise through several molecular pathways. The stratification of CRC is also based on molecular features like CIN, MSI, and CIMP.

One of the first studies focusing on molecular subtypes was that of Jass et al. [123]. The authors proposed five molecular subtypes of CRC, which were based on CIN and CIMP status. The basic stratification is shown in Table 1.

One year later, a similar classification based on genetic and epigenetic characteristics was introduced [127]. Except for typical molecular characteristics, this classification also included clinical, histological, and pathological features like localization, degree of differentiation, prediction of prognosis, and gender bias. A high methylation profile was described for two groups. Group 1 commonly shows *MLH1* methylation, *BRAF* mutation, and high MSI, and it is associated with a good prognosis, poor differentiation, elderly females, and proximal colon. Group 3 has similar characteristics, but these tumors are typically MSI-low or -stable, and the specific localization is the right-side colon.

In the study of de Sousa et al. [128], the authors classified 90 CRC patients into three subtypes. Two subtypes were effortless split up due to specific characteristics (CCS1 associated with CIN and left-sided, CCS2 associated mainly with MSI and right-sided), whereas the results for the CCS3 subtype were more controversial. For this reason, the authors focused on other molecular and clinical characteristics and described that this subtype is also characterized by promoter methylation of WNT target genes [129]. Patients classified into this subtype showed a worse prognosis—more than 50% of them developed a recurrence. Moreover, CCS3 patients with metastatic CRC were resistant to cetuximab independent of the *KRAS* mutation [128].

Kaneda and Yagi [120,130] developed “two-panel model” methylation markers for CRC classification. First, the authors suggested clustering CRC into three DNA methylation epigenotypes: low- (LME), intermediate- (IME), and high-methylation (HME) epigenotypes. In addition, methylation markers were classified into two other groups: Group 1 methylated specifically in HME that combines the most reported CIMP-related markers, and Group 2 methylated both HME and IME. With this approach, CRC can be properly classified: the first panel (*CACNA1G, LOX, SLC30A10*) extracts HME using Group 1 methylated markers and the second panel (*ELMO1, FBN2, THBD, HAND1* and *SLC30A10* again) divides the remaining IME and LME using Group 2 markers. The HME correlates with MSI-H and *BRAF* mutations, while IME correlates with *KRAS* mutations and shows a worse prognosis.

Nevertheless, this epigenotype stratification was further replaced by the CMS1 to CMS4 classification proposed by Guinney et al. [131].

**Figure 1:**
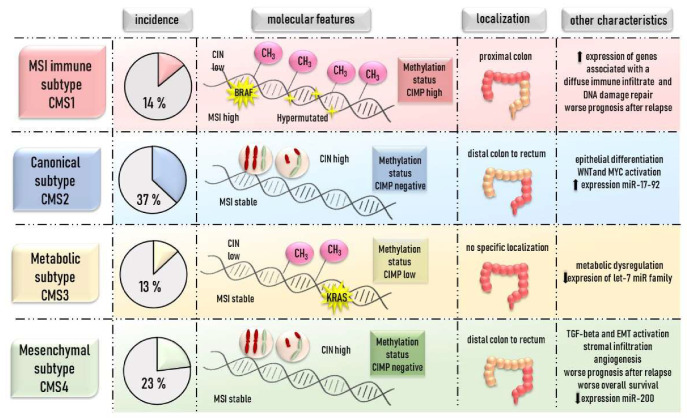
Consensus molecular subtypes (CMS) classification as proposed by Guinney et al. [131].

There have been a number of other studies defining a lot more CRC subtypes [132,133]. However, it was necessary to come up with some larger study and to establish a gold standard. It was done in 2015 when Guinney et al. [131] described four molecular CRC subtypes. The authors gathered the world’s largest evaluation of data of about 3443 CRC patients (stages II and III) and found out that 87% of CRC cases can fit into four specific groups. This molecular stratification was named as “consensus molecular subtypes” and established as CMS1 (MSI immune subtype), CMS2 (canonical subtype), CMS3 (metabolic subtype), and CMS4 (mesenchymal subtype). The summary of typical characteristics for these subtypes is shown in Figure 1. Briefly, high hypermethylation status is peculiar for CMS1, which is as well characterized by a hypermutated status, high MSI, *BRAF* mutation, increased expression of genes associated with a diffuse immune infiltrate, and worse prognosis. Intermediate level of gene hypermethylation is also observed in CMS3, for which there is a typical *KRAS* mutation and elevated multiple metabolic signatures. The prognostic effects of CMS subtypes in metastatic CRC are different from those observed in the early stages of CRC, where patients from CMS1 subtype displayed better relapse-free survival, and those in CMS4 subtype evinced shorter relapse-free survival [131]. However, after the assessment of the survival after relapse, the prognostic effects of CMS subtypes were similar between the early-stage CRC patients and the metastatic cohorts [131].

Despite many studies published in this field, the relationship between CIMP and molecular subtypes is still not entirely clear. In the last year, Fennell et al. [122] tried to distinguish molecular subtypes based on methylation as the first criterion and thus distinguished patients into 5 groups based on methylation profiles—high levels (CIMP-H1 and CIMP-H2), intermediate levels (CIMP-L1 and CIMP-L2), and low levels (CIMP-negative). It was depicted that CIMP-H1 is strongly correlated with the *BRAF* mutation, and CIMP-H2 is characteristic of the *KRAS* mutation. The authors showed that increasing methylation status correlates with increasing age, sex, and tumor location. Moreover, it was shown that methylation has a role in the progression of serrated neoplasia.

The inclusion of patients with distinct CRC molecular subtypes represents an essential start for clinical translation. The prognostic significance of CMS subtypes in both early and metastatic CRCs bolsters the fact that they could be used in the assessment of therapy responses and might aid the treatment choice.

Recently, Kwon et al. [134] demonstrated that molecular classification (CMS1–4) has a predictive value for the prognosis for stage III CRC patients treated with FOLFOX (folinic acid (leucovorin) “FOL”, fluorouracil (5-FU) “F”, and oxaliplatin “OX”) adjuvant chemotherapy. Del Rio et al. reported that the CMS4 subtype was enriched in FOLFIRI (folinic acid (leucovorin) “FOL”, 5-FU “F”, and irinotecan “IRI”) responders by the analysis of 143 CRCs [135]. In another study by Mooi et al. [136], CMS2 and possibly CMS3 tumors benefited from the addition of bevacizumab to first-line capecitabine-based chemotherapy, compared with other CMS groups. The mechanisms responsible for the interaction of CMS groups with bevacizumab treatment are not yet clear. However, it is remarkable that CMS2 and CMS3 tumors, which responded to bevacizumab, have similar characteristics of highly proliferative epithelial tumors, different from CMS1 and CMS4 that display high immune and stromal infiltration [137]. Okita et al. [138] showed that irinotecan-based therapy is highly effective for CMS4 patients.

CMS analyses in two other metastatic CRC trial cohorts, Cancer and Leukemia Group B (CALGB) 80405 and FIRE-3, have been recently reported [139,140]. In these randomized trials, patients with nonmutated *KRAS* tumors treated with cetuximab or bevacizumab in combination with first-line chemotherapy for metastatic CRC were studied. Lenz et al. [140] observed that CMS1 subtype patients treated with bevacizumab had a significantly longer overall survival (OS) in contrast to those treated with cetuximab, and CMS2 patients treated with cetuximab had a significantly longer OS than patients treated with bevacizumab. These best results were achieved in the subset of CMS1 patients with MSI-H tumors, while the CMS2 group was enriched for left-sided tumors that also responded well to cetuximab therapy in another study [141]. The authors also suggested that oxaliplatin, in combination with bevacizumab, may induce synergistic effects, resulting in marked clinical benefit for patients with CMS1 subtype tumors [128,139,140,142]. In contrast, Stintzing et al. [139] reported better OS after FOLFIRI plus cetuximab therapy versus FOLFIRI plus bevacizumab for CMS3 and CMS4 subtype CRC patients in the FIRE-3 study. Aderka et al. [143] hypothesized that the discrepancies monitored between these two trials, CALGB80405 and FIRE-3, can be due to the trial-specific sequence of chemotherapy plus targeted therapy. However, a recent study of Buchler et al. [144] showed no differences in OS between cohorts treated with first-line bevacizumab and second-line EGFRi (cetuximab or panitumumab) vs. the reverse sequence. Another difference between the data presented by the CALGB80405 and FIRE-3 groups was observed for CMS1 and MSI tumors. In the CALGB80405 study, the CMS1 subtype benefited from the bevacizumab treatment, while cetuximab-treated patients displayed worse outcomes [140]. The poor efficacy of cetuximab in the CMS1 subtype tumors was also noticed in the adjuvant PETACC8 trial. In this trial, patients with CMS1 subtype after the FOLFOX plus cetuximab therapy displayed shorter disease-free survival in comparison to FOLFOX administered alone [145]. This is in agreement that for patients with CMS1 subtype tumors, where oxaliplatin-based therapy combined with cetuximab may actually produce a harmful effect. Concerning the left-sided *KRAS* wild-type tumors that should be treated with an anti-EGFR agent in first-line treatment according to international guidelines [146,147], the longer OS induced by cetuximab treatment was predominantly evident in CMS3 and CMS4 subtypes in the FIRE-3 study. In the same study, FOLFIRI treatment with either cetuximab or bevacizumab in the CMS2 grouping had no predictive impact.

In short, the CRC classification by CMS subtypes could serve as a significant prognostic biomarker. However, from the clinical point of view, CMS does not seem to have superior value to routinely used clinical indication criteria for selecting patients for optimal treatment with either anti-EGFR or anti-VEGF agents. Overall, the CMS categorization provides a detailed insight into CRC etiology but currently still has no real impact on clinical decision-making.

## 8. Clinical Applications of DNA Methylation Profiling

Despite the recent progress in the identification of new cancer therapeutics, patients’ response rates to systemic therapy still remain low. The determination of prognostic and predictive biomarkers based on the DNA methylation profiles that can predict patient outcomes and therapeutic responses of advanced CRCs represents an important goal in CRC research. Here, we describe studies investigating the potential utility of methylated genes as predictive biomarkers.

The majority of CRCs (around 80%) evolve through the chromosomal instability pathway (CIN), whereas 10–15% are derived from the microsatellite instability (MSI) pathway that arises as a consequence of a deficient (d) DNA mismatch repair (MMR) system.

Over the last decade, extensive CRC research has suggested several molecular biomarkers, both of prognostic and predictive value. Although plenty of biomarkers have been extensively analyzed, very few of them were confirmed to be valid for the management of CRC, including defects in DNA mismatch repair (MSI phenotype) and *KRAS* and *BRAF* mutations. All CRC patients should be regularly tested for MMR and MSI status. Patients with CRCs greatly benefit from MSI testing as around 15% of them have deficient MMR tumors (dMMRs), while only 3–5% can be found in mCRCs.

In detail, patients with CRC stages II and III and dMMR after 5-FU based adjuvant therapy were associated with significantly lower tumor recurrence rates and improved survival rates compared with patients with proficient MMR cancers [148]. Overall, in patients with CRC stage II and dMMR, several studies have shown an insufficient benefit of 5-FU-based adjuvant chemotherapy. In patients with stage III disease, the predictive effect of MMR status for adjuvant chemotherapy still remains controversial [149].

Evrard et al. [150] observed that hypermutated dMMR/MSI mCRCs evince higher sensitivity to inhibitors of an immune checkpoint that stimulate cytotoxic T-cells to eliminate dMMR/MSI tumor cells. Additionally, Le et al. [151] concluded that patients with dMMR/MSI tumors greatly benefit from immunotherapy, regardless of the tumor type, with disease control rates of 80% and OS superior to three years in chemoresistant mCRC. On the other hand, the mCRC dMMR/MSI phenotype has been associated with worse prognosis and chemoresistance to standard treatment [150,152]. Recent studies have reported prolonged OS in dMMR/MSI mCRC after antivascular endothelial growth factor (anti-VEGF) treatment as compared with anti-EGFR treatment but without a change in survival conferred to chemotherapy regimen [152]. Finally, recent nonrandomized trials have pointed to the high efficacy of immune checkpoint inhibitors in dMMR/MSI chemoresistant mCRC as a reason for the high tumor mutational burden.

The most studied epigenetically suppressed gene, with an undeniable impact on CRC etiology, is the *hMLH1* hypermethylation. *hMLH1* encodes proteins involved in DNA mismatch repair (MMR), a system that works in a coordinated way to correct DNA mismatches in humans [5,153]. Several years ago, it was noticed that promoter *hMLH1* hypermethylation is associated with a mutator phenotype in sporadic MSI CRCs [154]. The determination of MSI in CRC patients is crucial for their clinical administration. MSI status is routinely assessed by immunostaining of MLH1 and/or by polymerase chain reaction (PCR) amplification of microsatellite sequences that are very stable among humans [155]. CRC patients with MSI display a better prognosis than MSS patients [156]. On the other hand, MSI patients are resistant to 5-FU therapy while being sensitive to oxaliplatin [157]. The study of Jover et al. [158] focused on the determination of CIMP status using the methylation status of five genes: *CACNAG1*, *SOCS1*, *RUNX3*, *NEUROG1*, and *MLH1*. CIMP-negative patients that received adjuvant 5-FU chemotherapy had significantly longer disease-free survival (DFS).

The immunogenic potential of MSI colorectal tumors has resulted in the development of immunotherapeutic approaches [159,160,161,162]. Immunomodulatory monoclonal antibodies, such as nivolumab and pembrolizumab, block programmed cell death protein 1 (PD-1), a negative regulator of T-cell activity, and thus enhance the anti-tumor response of T-cells [163]. Recently, it was observed that MMR status profited from therapy based on immune checkpoint blockade with pembrolizumab [151] and with nivolumab [164] in metastatic carcinomas. The positive effect of pembrolizumab was not restricted to MSI CRC and was also observed in metastatic carcinomas with MSI in various tissues of origin [151]. Based on these results, the food and drug administration (FDA) recently approved the use of pembrolizumab and nivolumab as a therapy choice for metastatic solid MSI tumors.

*MGMT* is a gene-encoding O^6^-methylguanine-DNA methyltransferase that repairs mutagenic adduct O^6^-methylguanine, formed by DNA alkylating agents. If these lesions are unrepaired, mutations (GC:AT transitions) arise during DNA replication. Epigenetic silencing of the *MGMT* gene has been associated with G > A mutations in *KRAS* and *TP53* genes [165]. Hypermethylation of *MGMT* has also been observed in adenomas and in the nonmalignant colonic mucosa of CRC patients, and it predicts worse prognosis in different carcinomas, but not in CRC. On the other hand, *MGMT* hypermethylation has been associated with better therapy response using alkylating agents [166,167]. In the study of Nagasaka et al. [168], the methylation status of the *MGMT* gene in tumor tissue from 116 CRC patients receiving adjuvant 5-FU based chemotherapy was determined. The *MGMT* hypermethylation was associated with better prognosis—patients had a lower chance of experiencing a recurrence after 5-FU based therapy. In addition, the promoter *MGMT* hypermethylation in plasma of CRC patients has been associated with better response to neoadjuvant therapy [169].

The prediction of therapy response based on the methylation status of *LINE-1* gene has also been indicated. In 155 CRC patients (stages II and III) treated with adjuvant 5-FU chemotherapy, *LINE-1* hypomethylation was associated with better survival [170]. However, the study of Kaneko et al. [171] described that patients with hypomethylated *LINE-1* showed a significantly worse response to FOLFOX-based chemotherapy compared to patients with hypermethylation of this gene. These result discrepancies could be partially explained by the composition of patients’ cohorts, as the study of Kaneko et al. [171] included stage IV patients.

Better response to treatment associated with gene hypermethylation was identified in the study of Jiang et al. [172], where the methylation status of the *WNT5A* gene was analyzed. The methylation level was significantly higher in patients that respond to 5-FU-based therapy. The vast majority of genes that are found to be hypermethylated are associated with a worse response to therapy. CRC patients with hypermethylated *NKX6.1*, *TFAP2E*, *IGFBP3*, and *HYAL2* genes did not profit from 5-FU-based chemotherapy [173,174,175,176].

A study focusing on irinotecan-based therapy, as described by Shimizu et al. [177], showed significantly shorter OS and chemoresistance for CRC patients with a methylated *BNIP3* gene. Hypermethylation of the *CDKN2A* gene (encoding protein p16) was associated with a worse response for CRC patients after FOLFOX chemotherapy [178,179]. Patients with no methylated *RASSF1A* gene had a better response to oxaliplatin-based therapy than patients with aberrant methylation [180].

Aberrant changes in DNA methylation are very promising as these tumor-derived changes might also be monitored in body fluids or stool specimens. Global hypo- and hypermethylation can also be assessed in circulating cell-free DNA isolated from the plasma of CRC patients, thus revealing a novel and low-invasive diagnostic biomarker that is also relevant for early-stage [181].

In summary, several predictive markers based on the DNA methylation profile have been published so far. However, none of these markers have been used in clinics. Studies focusing on the hypermethylation of the *MGMT* gene showed similar correlations with a good response to 5-FU-based therapy in CRC patients [168,169]. However, more recent, larger, and nonrandomized studies are still needed to confirm these outcomes. For stages II and III CRC patients with 5-FU-based adjuvant chemotherapy, Perez-Carbonell et al. [175] observed that *IGFBP3* hypomethylation was associated with longer overall survival and disease-free survival. However, survival was not affected in patients with *IGFBP3* hypermethylation.

The CIMP phenotype has also been studied for its predictive value but with conflicting results. CRC patients with negative or low CIMP and with 5-FU- or oxaliplatin-based chemotherapy displayed better prognosis, while patients with positive or high CIMP was associated with better outcomes after chemotherapy in another study [158,182]. Additionally, no association between CIMP status and CRC chemotherapy has been monitored in other studies [178,183].

One of the most studied methylated genes in CRC in body fluids is *SEPT9*. Septins are GTP-binding proteins that play a role in the regulation of cell cycle and cytokinesis [184]. In a liquid biopsy approach, many studies have proven that *SEPT9* can distinguish CRC patients from healthy controls and thus suggested its potential to use this gene as a diagnostic biomarker [185,186,187]. Other studies have also shown its prognostic potential [188]. Unfortunately, there have not been many studies focusing on therapy response prediction. In the study of Bhangu et al. [189], the methylation status of *SEPT9*, *DCC*, *BOLL*, and *SFRP2* genes was established in repeated plasma samples from 34 patients with CRC liver metastasis that had undergone neoadjuvant chemotherapy. The authors observed the dynamic changes in methylated *SEPT9* and *DCC* genes during neoadjuvant chemotherapy. Good responders to neoadjuvant therapy methylation levels decreased during the first 2 cycles.

Monitoring of the plasma levels of mSEPT9 in patients with CRC during treatment seems to be very promising, as the methylated SEPT9 gene has been implicated as a biomarker for colorectal cancer. Currently, one clinical trial (NCT03334890) is already ongoing to assess the performance of mSEPT9 in assessing the surgical therapeutic effect of CRC.

The studies analyzing the methylation profile as a predictive biomarker, described in this paragraph and others not mentioned above, are summarized in Table 2.

Besides, many CRC prognostic markers based on the DNA methylation status have been published in the literature so far. To date, none of these markers have been used in clinical practice. Recently Draht et al. [190] and Ma et al. [191] comprehensively reviewed the published prognostic methylation markers for CRC and marked the most promising ones. Studies focusing on the hypermethylation of the *IGFBP3* gene have shown correlations with a poor prognosis in CRC patients [175,192,193].

The *CDKN2A* hypermethylation was also associated with poor prognosis in CRC patients [182,194,195,196,197]. Interestingly, conflicting results for association with patient survival were observed for *MLH1* promoter hypermethylation [198,199,200,201,202]. Many authors have hypothesized that the analysis of all CRC patients as an entity might obscure the real potential of identified biomarkers.

## 9. DNA Methyltransferase Inhibitors

The DNA methyltransferases (DNMTs), an enzyme that specifically catalyzed DNA methylation, have an important role in the regulation of gene expression, with implications in many diseases, including cancer. The recent research goals lay in the development of therapeutic DNMT inhibitors [204,205]. Inhibitors of DNMT can be divided according to their mechanism of action into two groups: nucleoside analogs that incorporate into DNA, and non-nucleoside inhibitors that interact directly with DNMTs. Both actions lead to the formation of a covalent complex with DNMTs.

Several global DNMT inhibitors, i.e., inhibitors targeting all the DNMTs, have been used in CRC therapy within clinical trials.

### 9.1. Azacitidine

**Figure 2:**
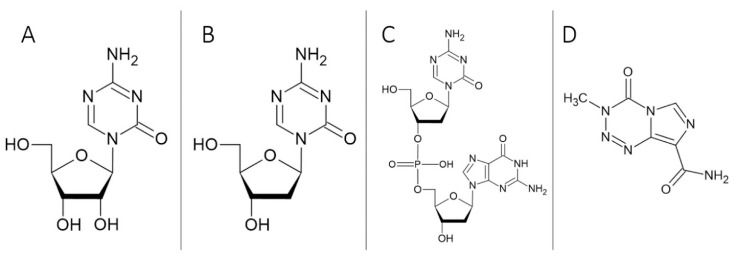
(**A**) Azacitidine, (**B**) decitabine, (**C**) guadecitabine, (**D**) temozolomide.

Azacitidine (5-AZA; Figure 2A), a chemical analog of pyrimidine nucleoside of cytidine, differs by the presence of nitrogen at position 5 of the pyrimidine ring. This molecule was first characterized as a DNMT inhibitor. As a chemotherapeutic agent used in the treatment of cancer, especially for myelodysplastic syndrome and acute myeloid leukemia, it was approved by the FDA in 2004 and by the European Medicines Agency (EMA) in 2002. In clinical use, it is known under the name Vizada [206].

Once 5-AZA is transported into the mammalian cell, 5-AZA is subsequently phosphorylated three times by the serial action of uridine-cytidine kinase and then ribonucleotide reductase to its activated form, azacitidine triphosphate [207,208]. The mechanism of 5-AZA cytotoxicity is through the incorporation into RNA, causing the inhibition of protein synthesis and incorporation into DNA, leading to DNMT depletion. This also supports normal cell growth and cell differentiation by demethylation and the re-expression of tumor suppressor genes [205].

### 9.2. Decitabine

Decitabine (5-aza-2′-deoxycytidine, DAC; Figure 2B) was discovered together with 5-AZA. DAC is also one of the analogs of cytidine and is very similar to 5-AZA. However, it incorporates only into DNA due to a deoxyribonucleoside (2′-deoxy-5-azacytidine) core. DACs have been reported to be more specific and less toxic than 5-AZA, but both are useful inhibitors of DNA methylation even at low concentrations [209,210]. The knowledge of DAC and 5-AZA cytotoxic effects has strong clinical implications, as these drugs cause DNA damage and protein synthesis disruption at higher concentrations [211,212].

As a drug, it is known under the name Dacogen and was approved by the FDA in 2006 and, in the same year, also by the EMA. The application of DAC is the same as with 5-AZA for myelodysplastic syndrome and acute myeloid leukemia [213]. Nevertheless, 5-AZA has demonstrated better improvement of OS than decitabine [214].

### 9.3. Guadecitabine

Guadecitabine (SGI-110, S110; Figure 2C) is a unique hypomethylating prodrug of the second generation. SGI-110 is a dinucleotide consisting of DAC connected through a phosphodiester bond to deoxyguanosine that provides protection from cytidine deaminases and, thus, has a prolonged plasma half-life. SGI-110 was designed with better pharmacokinetics and metabolic properties as a replacement for hypomethylating agents such as 5-AZA and DAC [215]. Its lower toxicity and better biological stability were determined in comparison to DAC in vivo in tumor-free nude mice [215]

### 9.4. Temozolomide

Temozolomide (TMZ; Figure 2D) is an oral prodrug developed in the 1980s in Great Britain [216]. Thanks to its small size (194 Da), it is well absorbed in the small intestine and penetrates easily through the blood–brain barrier. Tumor or nonmalignant cells can be resistant to TMZ via high levels of *MGMT*, which repairs the O^6^-methylguanine (O^6^-meG) by transferring the methyl group from the O^6^-meG to its own cysteine residue. Nevertheless, the cytotoxicity of TMZ could be enhanced by promoter hypermethylation of the *MGMT* gene [217].

TMZ was approved by the FDA in 1999 for the treatment of patients with primary brain tumors [217] and in 2005 for the concomitant treatment of patients with newly diagnosed glioblastoma with radiotherapy. In clinical use, TMZ is known under the name Temodar [218]. In 2010, Temodar’s generic version, named Teva, was also approved by EMA.

## 10. Methylation-Based Therapies

DNMT inhibitors have been studied as the treatment of CRC patients, especially those with evidence of higher levels of DNA methylation.

Demethylation drugs AZA and DAC are synthetic cytosine analogs, which have already been approved for the treatment of acute myeloid leukemia and myelodysplastic syndrome [5,219]. Inhibition of DNMTs by DAC and 5-AZA depends on the DNA replication, as both AZA and DAC can be incorporated into DNA to form covalent bonds with DNMTs and therefore block the DNMT methylation activity. Preclinical studies have shown that DNMT inhibition by AZA or DAC induces apoptosis and reduces the growth of CRC cells. The cytotoxic effect of DAC and AZA has been demonstrated in vitro as well as in xenograft models [220].

In Table 3, seventeen ongoing or completed clinical trials exploring the effect of AZA, DAC, SGI-110, or TMZ in the treatment of CRC patients are listed. These trials are focused on early phases (I and II), with the aim of identifying the outcomes of the utilization of these drugs as single-agent therapy or in combination with immunotherapy or cytotoxic chemotherapy. Of these, eight have been completed, two have been terminated (NCT01882660 due to slow recruitment and NCT00423150 due to low response rate), and the rest are still ongoing or recruiting. However, most of these studies did not show a clear connection between the level of induced demethylation and clinical response, although in most cases, the drugs were well tolerated.

Most of the trials were limited by the exclusion of initial patient stratification according to their methylation profile. The stratification of patients that would benefit from the recurring expression of the already repressed genes by DNMT inhibitors could be promoted by the identification of driver or passenger methylation issues [5]. Special attention is focused on methylation in tumors with epigenetic silencing of hMLH1 (involving in most of the CIMP-high CRCs). When the mutator phenotype is released, numerous mutations might accumulate in MSI-target cancer genes, which largely influence the tumor phenotype [221]. Mutations in the cancer driver genes cannot be repaired by this hMLH1 re-expression; nevertheless, this process could be helpful for increasing the sensitivity to cytotoxic chemotherapy [222].

## 11. Conclusions

Despite current progress in the improvement and invention of drugs with anticancer activity, the current systemic therapy for CRC is far from optimum due to limited efficacy in the unselected patient population. This highlights the necessity to identify new biomarkers that can determine the patient’s response to a specific treatment.

The study of DNA methylation is in the foreground, and it has been shown that methylation status can predict patients’ response to treatment. In detail, methylation of *MGMT* and *IGFB3* genes and CIMP status are considered for their predictive value in 5-FU based-chemotherapies. DNA methylation markers may thus improve treatment strategy and could even be used for screening in the future. In addition, DNA methylation biomarkers may facilitate the development of precision medicine.

The main problem with the use of global and specific DNMT inhibitors is their excessive demethylating effect that leads to the reactivation of tumor suppressor genes as well as oncogenes. A new approach should lie in the development of such DNMT inhibitors that might target the specific interaction, utilizing DNMT targets and leading thus to antitumorigenic phenotypes with no modification of oncogenes by methylation (i.e., oncogene inhibition). Currently obtained results exploiting this concept are encouraging.

In addition, the combination of methylation-based therapies with standard chemotherapeutic drugs shows both additive and synergistic effects. However, many epigenetic modification-targeting drugs such as demethylating agents 5-AZA and DAC are inherently and highly toxic. Another problem with DAC and 5-AZA is their rapid elimination in plasma; this problem circumvented by the development of their derivatives for longer half-life, such as SGI-110.

Several global projects have been developed to facilitate our understanding of how genetic and epigenetic mechanisms regulate gene expression, such as the Encyclopedia of DNA Elements (ENCODE, 2003), The Cancer Genome Atlas (TCGA, 2006), and International Cancer Genome Consortium (ICGC, 2008), the Consortium for Epigenomics Mapping of the National Institutes of Health (2008), and the European Community Initiative BLUEPRINT (2011). Using next-generation sequencing approaches, these programs have helped to identify epigenomic differences between healthy and pathological states. Epigenomic profiling has significantly improved our understanding of complex human diseases, such as cancer. The International Consortium for Human Epigenome (IHEC, 2010) [232] was established to develop reference maps of at least 1000 epigenomes for health- and disease-related cell states [233] and to publish data to boost clinical applications.

Considerable efforts are currently being made to identify biomarkers associated with therapy response and thus discriminate against patients who will benefit from chemotherapy. The identification of these biomarkers is crucial for individualized treatment strategies in all CRC patients. The identification of these biomarkers might stratify patients into groups with specific treatment and promote the shift from a universal approach to precision medicine. These biomarkers should be able to distinguish responders from nonresponders with relative ease and low cost and to be non-invasive to increase screening acceptability. DNA methylation markers can improve treatment strategy in the future and facilitate the development of precision medicine. We believe that these changes can be tracked non-invasively in ctDNA released into plasma during the administration of chemotherapy. The sequential methylation mapping in serial blood specimens might contribute to the improvement of therapeutic strategies in CRC.

## Figures and Tables

**Table 1:** Molecular stratification by Jass et al. [123].

	Molecular Subtypes				
Characteristics	Group 1	Group 2	Group 3	Group 4	Group 5
MSI status	high	stable–low	stable–low	stable	high–stable
Methylation status	high (*MLH1*)	high (*MLH1*)	low (*MGMT*)	negative	negative
Mutations	*BRAF*	*BRAF*	*KRAS*	*APC*	-
Origin	serrated polyps	serrated polyps	adenomas serrated polyps	adenomas	adenomas
% of cases	12	8	20	57	3

**Table 2:** Summary of studies focusing on the predictive potential of methylation profile.

Origin of the Study	Source	Number of Patients	Gene	Method	Treatment Relevance	Reference
Japan	tissue	116	*MGMT*	MS-PCR	hypermethylation predicts good response to 5-FU-based chemotherapy	[168]
Japan	tissue	155	*LINE-1*	MS-PCR	hypomethylation predicts good response to 5-FU chemotherapy	[170]
Japan	tissue	112	*BNIP3*	qPCR	presence of methylation is associated with worse response to IRI based chemotherapy	[177]
Spain	tissue	302	*CACNAG1* *SOCS1* *RUNX3* *NEUROG1* *MLH1*	pyrosequencing	patients with CIMP+ status do not benefit from 5-FU chemotherapy	[158]
Germany	tissue	74	*TFAP2E*	qPCR	hypermethylation is associated with worse response to 5-FU chemotherapy	[174]
Korea	tissue	322	*NEUROG1* *CDKN2A*	Methylight	concurrent hypermethylation of both genes is associated with recurrence after adjuvant FOLFOX	[178]
USA	tissue	425	*IGFB3*	bisulfite pyrosequencing	hypermethylation predicts worse response to 5-FU chemotherapy	[175]
China	plasma	34	*MGMT*	MS-PCR	hypermethylation is associated with a better response to chemoradiotherapy based on capecitabine and OX	[169]
Germany	tissue	232	*HYAL2*	MALDI-TOF mass spectrometry	hypermethylation predicts worse response to 5-FU chemotherapy	[176]
Japan	tissue	40	*LINE*-1	Methylight	hypomethylation predicts bad response to FOLFOX	[171]
Korea	tissue	49	*CDKN2A*	pyrosequencing	hypermethylation predicts worse response to chemotherapy based on 5-FU and IRI	[179]
China	tissue	126	*WNT5A*	MS-PCR	hypermethylation is associated with better response to 5-FU chemotherapy	[172]
China	tissue	108 CRC 78 HC	*RASSF1A*	MS-PCR	presence of methylation is associated with better response to OX based chemotherapy	[180]
Austria	plasma	34	*SEPT9* *DCC*	qPCR	tool for early response assessment in patients receiving neoadjuvant chemotherapy	[189]
China	tissue	151	*NKX6.1*	MS-PCR	presence of methylation is associated with worse response to 5-FU chemotherapy	[173]
Korea	tissue	102	*CHFR*	MS-PCR	Hypermethylation is associated with good response to chemotherapy based on IRI	[203]

5-FU: 5-fluorouracil, IRI: irinotecan, CIMP: CpG island methylator phenotype, OX: oxaliplatin, FOLFOX: folinic acid + fluorouracil + oxaliplatin, MS-PCR: methylation-specific PCR, qPCR: quantitative PCR.

**Table 3:** Clinical trials on hypo- and hypermethylating chemotherapeutic drugs used in colorectal cancer treatment.

Clinical Trial	Status/ Durance	Origin of Study	Condition	Number of Patients	Drug	Reference
NCT01105377	Completed/ 2010–2014	US	Recurrent CC, Recurrent RC, Stage IV CC, Stage IV RC	47	Azacitidine	[223]
NCT02959437	Completed/ 2017–2020	US, UK, Spain	Solid Tumors, Advanced Malignancies, Metastatic Cancer	70	Azacitidine	-
NCT01193517	Completed/ 2010–2016	US	CRC	26	Azacitidine, Azacitidine MTD	-
NCT02260440	Completed/ 2015–2017	US	mCRC	31	Azacitidine	[224]
NCT02811497	Active, not recruiting/ 2016–2022	Canada	MSS CRC, Platinum-Resistant Epithelial Ovarian Cancer Type II, Estrogen Receptor-Positive and HER2-Negative Breast Cancer	28	Azacitidine	-
NCT02316028	Completed/ 2014–2017	Belgium	Liver Metastasis, CRC	11	Decitabine	[225]
NCT00879385	Completed/ 2009–2013	US	CRC (wild-type KRAS mCRC)	21	Decitabine	[226]
NCT01896856	Completed/ 2013–2019	US, Netherlands	Previously Treated mCRC	96	SGI-110 (Guadecitabine)	[227]
NCT01966289	Active, not recruiting/ 2013–2020	US	mCRC	18	SGI-110 (Guadecitabine)	-
NCT03576963	Recruiting/ 2020–2023	US	CRC CIMP, MSS mCRC, Refractory CC, CRC Stage IVA, Stage IVB, and Stage IVC	45	Guadecitabine	-
NCT03519412	Recruiting/ 2019–2022	Italy	CRC, MSI	348	Temozolomide	-
NCT01051596	Completed/2009–2013	US	CRC	75	Temozolomide	[228]
NCT03832621	Recruiting/ 2019–2022	Italy	mCRC	100	Temozolomide	-
NCT04166435	Active, not recruiting/ 2020–2022	US	CRC	30	Temozolomide	-
NCT02414009	Unknown/ 2014–2017	Italy	mCRC	82	Temozolomide	[229], [230]
NCT01882660	Terminated/2013–2018	Netherland	CC	88	Decitabine	-
NCT00423150	Terminated/2007–2017	UK	CRC, Head and Neck Neoplasm Carcinoma, Non-Small-Cell Lung, Esophageal Neoplasm	86	Temozolomide	[231]

CRC: colorectal cancer, mCRC: metastatic CRC, CC: colon cancer, RC: rectal cancer, CIMP: CpG island methylator phenotype, MSI: microsatellite instability, MSS: microsatellite stable.

## Abbreviations

5-AZA	azacytidine
5-FU	5-fluorouracil
5-mC	5-methyl-cytosine
CALGB	Cancer and Leukemia Group B
CC	colon cancer
CGIs	CpG islands
CIMP	CpG island methylator phenotype
CIMP-L	CpG island methylator phenotype low
CIN	chromosomal instability
CMS	colorectal molecular subtype
CRC	colorectal cancer
DAC	decitabine
DFS	disease-free survival
DNMT	DNA methyltransferases
EMA	European Medicines Agency
FDA	Food and drug administration
FOL	folinic acid
HNPCC	hereditary nonpolyposis colorectal cancer
IRI	irinotecan
mCRC	metastatic CRC
MMR	DNA mismatch repair
MSI	microsatellite instability
MS-PCR	Methylation-specific PCR
MSS	microsatellite stable
O^6^-meG	O^6^-methylguanine
OS	overall survival
OX	oxaliplatin
PCR	polymerase chain reaction
PD-1	programmed cell death protein 1
qPCR	quantitative PCR
RC	rectal cancer
SGI-110	guadecitabine
TMZ	temozolomide
TSS	transcriptional start sites
